# A qualitative exploration of COPD lived experience, giving insight into symptom profile determinants and experiences of medical and non-medical interventions

**DOI:** 10.1080/17482631.2026.2755682

**Published:** 2026-11-07

**Authors:** Emma Sharpe, Samuel Grimwood, Amy Baraniak, Mark A Faghy

**Affiliations:** a College of Science and Engineering, University of Derby, Derby, UK; b College of Health and Humanities, University of Derby, UK; c School of Sport, Exercise and Health Sciences, Loughborough University, UK

**Keywords:** Chronic obstructive pulmonary disease (COPD), symptom burden, qualitative research, self-management, reflexive thematic analysis

## Abstract

**Purpose:**

Chronic obstructive pulmonary disease (COPD) is characterised by a substantial symptom burden, including reduced physical activity, impaired sleep, diminished quality of life, and psychological distress. Despite established treatments, many individuals continue to experience persistent symptoms and functional limitations. This study aimed to explore symptom burden and how individuals manage COPD in daily life.

**Methods:**

Fifteen UK-based adults with a confirmed diagnosis of COPD (mean age 70 years, *n* = 10 females) participated in semi-structured interviews. Participants were recruited through clinical databases, research registries, and community support networks. Interviews were transcribed verbatim and analysed using inductive reflexive thematic analysis supported by NVivo.

**Results:**

Three themes were identified: (1) Interconnected symptom experiences, (2) Navigating treatment uncertainty and burden, and (3) Becoming an active manager of COPD. Breathlessness was described as the most debilitating symptom, closely interconnected with reduced functional capacity, and restricted social participation. Participants reported challenges with medication, including frequent treatment changes and limited perceived effectiveness. Self-management was central to daily life, with individuals employing pacing strategies, assistive devices, and self-directed learning.

**Conclusions:**

COPD is experienced as a complexcondition shaped by interconnected symptoms and self-management demands. These findings highlight a need for responsive, patient-centred approaches to COPD care.

## Introduction

Chronic obstructive pulmonary disease (COPD) is characterised by persistent airflow limitation and progressive respiratory decline and remains a leading cause of morbidity and mortality worldwide (May & Li, 2015). Beyond its primary pathophysiological effects, COPD generates a substantial secondary symptom burden driven by deteriorating lung function and chronic dyspnoea (Grimwood et al., 2025; Miravitlles & Ribera, 2017). These secondary manifestations include marked reductions in physical activity, impaired sleep architecture, diminished health-related quality of life, and elevated rates of psychological morbidity, particularly depression and anxiety (Panagioti et al., 2014; Siraj, 2025). Although multiple pharmacological therapy classes are available for COPD management, including bronchodilators and inhaled corticosteroids, symptom control remains an issue for a high proportion of patients (Hanania & Donohue, 2007; Tashkin & Strange, 2018).

The therapeutic objectives of COPD pharmacotherapy include attenuation of dyspnoea, prevention of exacerbations, and overall improvement in health status (MacLeod et al., 2021). Nevertheless, as the disease progresses, significant and irreversible impairment in lung function remains expected (Siafakas et al., 1995). Despite guideline-directed therapy, many individuals continue to report persistent symptomatology and functional limitations (Elsayed et al., 2026; Lewthwaite & Takemura, 2026). This indicates ongoing unmet clinical needs in relation to symptom management and exacerbation reduction (Halpin et al., 2017; MacLeod et al., 2021). Importantly, no pharmacological intervention has been shown to arrest or reverse the long-term decline in lung function (Wang et al., 2020), necessitating integration of additional non-pharmacological strategies.

Pulmonary rehabilitation (PR) remains the most established non-pharmacological intervention, offering benefits across exercise capacity, symptom burden, and quality of life (Hindelang et al., 2020). However, clinical utilisation remains limited by challenges relating to referral practices, programme accessibility, patient uptake, and service provision (Mulhall & Criner, 2016). Consequently, many individuals may not access PR until symptom burden is already substantial, potentially limiting opportunities to maximise benefit. Patients may also depend on assistive devices such as rollators for mobility support and perching stools to facilitate activities of daily living (Sewell, 2018). In the advanced stages of disease, some patients perceive their condition as irreversible and may consequently disengage from available clinical and functional support services (Seamark et al., 2004).

Obtaining a comprehensive understanding of patients' lived experiences is clinically important to identify factors contributing to persistent symptom burden despite multimodal management. This includes examining patients’ perceptions of pharmacological effectiveness, their daily medication practices, and the compensatory strategies employed when symptoms remain inadequately controlled. Such insights may reveal additional drivers of symptom distress, unrecognised barriers to self-management, and unmet clinical needs not addressed within current care pathways (Russell et al., 2018). Although existing evidence suggests that combined pharmacological and non-pharmacological approaches should offer meaningful symptom relief (ur Rehman et al., 2020), patients’ real-world experiences may not reflect this theoretical benefit.

Previous qualitative studies have demonstrated that COPD can adversely affect quality of life, social participation, emotional wellbeing, and self-management behaviours. However, much of the existing qualitative literature has explored these issues independently, with less attention given to how symptom experiences, treatment challenges, and self-management strategies interact as part of a broader lived experience. Understanding these interrelationships may provide important insight into why substantial symptom burden persists despite engagement with both pharmacological and non-pharmacological interventions. Accordingly, there remains a need for further qualitative research that explores how individuals experience and navigate these interconnected aspects of COPD in everyday life.

The need to focus on patient experiences is underpinned by health psychological models that emphasise the active role of individuals in interpreting and managing chronic illness. Leventhal’s Common-Sense Model of Self-Regulation proposes that patients form cognitive and emotional representations of their illness, which shape how they perceive symptoms and guide the selection of coping strategies, including both pharmacological and non-pharmacological approaches (Leventhal et al., 2016). Similarly, Symptom Management Theory conceptualises symptom experience as a dynamic process involving perception, evaluation, and response, highlighting how individuals interpret symptom burden and enact strategies to manage it (Dodd et al., 2001). These perspectives are situated within a broader biopsychosocial framework, which recognises that the experience of COPD is not solely determined by physiological impairment but is also influenced by psychological factors such as beliefs, coping, and self-efficacy, as well as social and contextual factors (Moos, 1984).

Together, these frameworks provide a useful conceptual context for understanding how individuals interpret, respond to, and manage chronic symptoms. Importantly, these frameworks informed the broader conceptual framing and interpretation of the study rather than guiding data collection, coding, or theme development. The analysis itself remained inductive and grounded in participants' accounts.

The present study also builds on our prior work, which examined the impact of COPD on quality of life and patients’ readiness to engage with interventions (Grimwood et al., 2026), by shifting focus towards the processes underpinning symptom experience and management. Specifically, this study aimed to characterise more explicitly the symptom burden associated with COPD by examining how patients experience and manage their symptoms through both pharmacological and non‑pharmacological interventions. In contrast to previous work, which has tended to examine symptom burden, treatment experiences, or self-management behaviours separately, this study sought to explore how these domains intersect within participants' everyday lives. In doing so, it integrates subjective symptom experience with the behavioural responses used to manage them, providing a more process-oriented and patient-centred understanding of COPD symptom burden and self-management. We therefore sought to understand how people living with COPD experience and manage symptom burden in everyday life, and how treatment experiences and self-management strategies shape their experiences.

## Methods

### Participants

Following ethical approval from the College of Science and Engineering Research Ethics Committee at the University of Derby (approval number ETH2021‑0357), fifteen participants with a confirmed diagnosis of COPD were recruited. This study was conducted in accordance with the ethical principles outlined in the Declaration of Helsinki. Participants provided written informed consent and were identified through three routes: an existing database of individuals with clinically confirmed COPD (*n* = 6); a database of previous research participants who had consented to be contacted about future studies (*n* = 6); and community-based recruitment via COPD support groups on social media and the Asthma+Lung UK network (*n* = 3). All participants resided in the United Kingdom.

Participants were eligible if they: (1) were aged 18 years or older; (2) had received a clinical diagnosis of COPD; (3) resided within the United Kingdom; (4) were able to participate in an English-language interview; and (5) provided informed consent. Individuals were excluded if they did not have a confirmed COPD diagnosis, were unable to participate in an interview, or were unable to provide informed consent. Purposive sampling was employed to recruit individuals with lived experience of COPD from a range of geographical locations and disease durations. Given the qualitative and experiential aims of the study, sample adequacy was judged according to informational power, whereby recruitment continued until sufficient depth and richness of data had been obtained to address the research objectives.

The sample was predominantly female (*n* = 10) with a mean age of 70 years. Half were married (*n* = 8), all identified as White British, and most were retired (*n* = 12). The majority had a primary diagnosis of emphysema and had been living with COPD for more than seven years (*n* = 10). In terms of pharmacological management, almost all participants (*n* = 12) were prescribed short-acting bronchodilators, half used long‑acting bronchodilators, and over half (*n* = 9) were prescribed inhaled corticosteroids. Most participants (*n* = 11) reported previous engagement with pulmonary rehabilitation. Comorbidity burden was common, with half of the sample reporting two comorbid conditions, most frequently hypertension and asthma. A detailed summary of participant characteristics is provided in Table I.

**Table I.** Participant characteristics and self-reported COPD-related clinical information.

Pseudonym	Age	Self-reported COPD diagnosis	Duration since diagnosis	Location	Smoking status	Pack years	Comorbidities	Current COPD treatment
Sue	71	Chronic bronchitis	10 years+	East Midlands	Ex Smoker	28.5	Heart Failure/Heart Disease, Asthma, Sleep Apnoea, Anaemia	Inhalers: Salbutamol, Tiotropium, Steroid Tablets: Theophylline, Steroid, Antibiotics Other treatment: Nebulised medicine (NM)
Patrick	70	Emphysema	7–10 years	South East	Ex Smoker	67.5	Heart Failure/Heart Disease	Inhalers: Salbutamol, Tiotropium
Melissa	66	Emphysema	5–7 years	East Midlands	Ex Smoker	45	Heart Failure/Heart Disease, Cancer	Inhalers: Salbutamol, Tiotropium, Steroid Tablets: Carbocisteine Steroid, Antibiotics Other treatment: NM, Ambulatory oxygen therapy (AOT)
Jane	60	Emphysema	10 years+	South East	Never Smoked	–	–	Inhalers: Terbutaline, Steroid
Malcolm	79	Emphysema	10 years+	West Midlands	Ex Smoker	30	Asthma	Inhalers: Steroid Other treatment: NM
Jacqueline	72	Don’t know	5–7 years	East Midlands	Ex Smoker	13	Hypertension, Asthma	Inhalers: Salbutamol, Tiotropium
Aimee	73	Emphysema	10 years+	South West	Ex Smoker	43	Hypertension, Diabetes, Asthma, Osteoporosis, Anaemia	Inhalers: Salbutamol, Aclidinium, Steroid Tablets: Theophylline, Carbocisteine, Antibiotics Other treatment: AOT
Pauline	77	Emphysema	10 years+	London	Ex Smoker	40	Rheumatic disease, Rhinitis	Inhalers: Tiotropium, Steroid Other treatment: AOT
Pat	65	Emphysema	5–7 years	South East	Ex Smoker	11.25	–	Inhalers: Salbutamol Tablets: Carbocisteine, Steroid, Antibiotics
Yvonne	69	Emphysema	7–10 years	Yorkshire & Humber	Ex Smoker	40	Hypertension, Asthma	Inhalers: Tiotropium, Steroid
Teresa	75	Chronic bronchitis	7–10 years	North West	Ex Smoker	36.25	Hypertension, Asthma, Osteoporosis, Stroke	Inhalers: Salbutamol, Tiotropium, Steroid Tablets: Theophylline
Tracy	76	Emphysema	7–10 years	East Midlands	Ex Smoker	50	Hypertension, Cancer	Inhalers: Salbutamol, Tiotropium, Glycopyronium Tablets: Carbocisteine Other treatment: IAOT
Suzanne	61	Don’t know	2–5 years	East Midlands	Never Smoked	–	Rhinitis	Inhalers: Salbutamol, Steroid
Marcus	79	Don’t know	5–7 years	Yorkshire & Humber	Never Smoked	–	Hypertension	Inhalers: Salbutamol, Glycopyronium
Sophie	67	Emphysema	7–10 years	North West	Ex Smoker	7.5	Hypertension, Asthma	Inhalers: Salbutamol

COPD diagnosis/descriptions (e.g., emphysema, chronic bronchitis, or “don't know”) reflect participant self-report during interview and were not verified against medical records. Smoking years are reported as pack-years. Medication categories are presented as reported by participants. AOT = ambulatory oxygen therapy; NM = nebulised medication; COPD = chronic obstructive pulmonary disease.

Objective measures of COPD severity (e.g., GOLD stage, spirometry data, or FEV1 values) were not collected as part of this study; therefore, disease severity was not considered during sampling or analysis (Figure 1).

**Figure 1.**
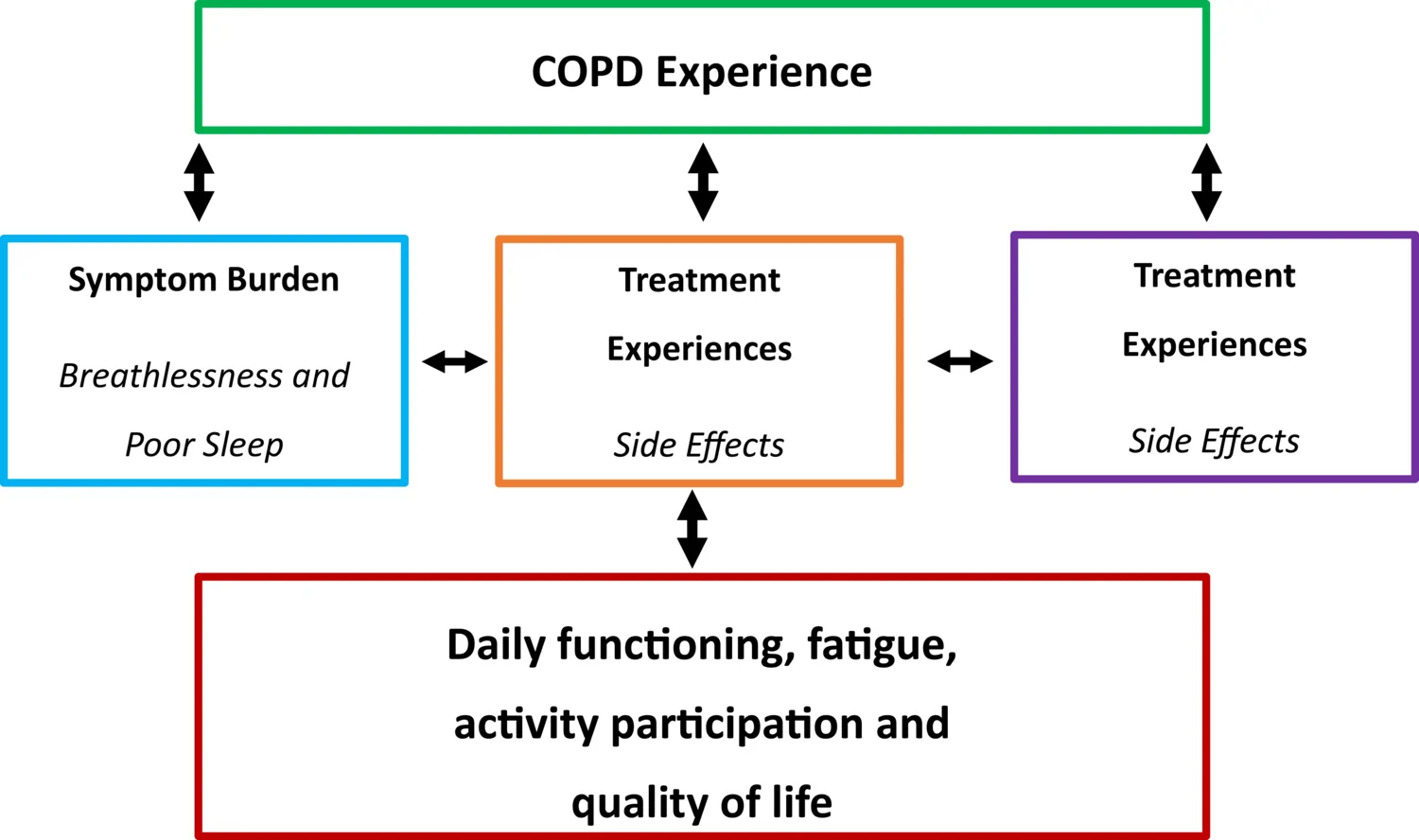
Conceptual model illustrating the interrelationships between symptom burden, medication experiences, and self-management in shaping the lived experience of COPD.

### Study design and procedure

Following informed consent and the scheduling of a mutually convenient interview time, a sixty-minute semi-structured interview was conducted online (via MS Teams) to elicit detailed, patient-centred accounts of symptom experience and disease management. This method enables flexible, in‑depth exploration of patient perspectives and facilitates access to experiential information that structured questionnaires cannot capture (Kakilla, 2021). The interview schedule was developed by the research team following a review of literature relating to COPD symptom burden, treatment experiences, and self-management. The guide was piloted internally by researchers experienced in qualitative interviewing, and no substantive changes were required. Questions explored symptom experiences, medication use, self-management strategies, interactions with healthcare services, and perspectives on future treatments and research. The schedule remained consistent throughout data collection, although interviewers used follow-up prompts flexibly to explore participant responses in greater depth. All interviews were recorded and transcribed verbatim, and all transcriptions were checked with ES and MF. Reporting of the study was informed by the Standards for Reporting Qualitative Research (SRQR) and the Reflexive Thematic Analysis Reporting Guidelines (RTARG).

### Analytic strategy

Reflexive thematic analysis (Clarke & Braun, 2017) was used to identify, analyse, and interpret patterns of meaning across the dataset. SG, who conducted all interviews, undertook an iterative analytic process involving data familiarisation, inductive coding, and theme development. Throughout the analysis, SG engaged reflexively with the data to support the generation of thoughtful, meaningful interpretations. Themes were developed inductively, reviewed, and refined to capture patterns of shared meaning across participants' accounts. Consistent with reflexive thematic analysis, themes were understood as interpretive outputs generated through the researchers' engagement with the data rather than entities that existed within the data awaiting discovery. This approach was chosen to allow participants’ perspectives to shape the analytic process, enabling rich, nuanced insights to emerge without being constrained by predefined theoretical assumptions. Although health psychological frameworks are discussed within the introduction, these informed the broader interpretation of findings rather than the coding process itself. Theme development remained inductive and grounded in participants' accounts.

SG carried out the primary analysis, including coding, naming, and finalising candidate themes. AB contributed as a critical reader during theme development, providing opportunities to challenge assumptions, explore alternative interpretations, and deepen analytic reflection. NVivo (version 15) was used to support data organisation, coding, and retrieval of extracts throughout the analytic process. Candidate themes developed by SG were discussed with AB, ES, and MF as part of an ongoing reflexive process. These discussions were used to explore alternative interpretations, challenge assumptions, and enhance analytic depth rather than to establish coding agreement or inter-rater reliability. This iterative movement between coding, thematic mapping, and reflexive discussion strengthened interpretive rigour and supported a richer understanding of participants' experiences.

### Reflexivity

The researchers acknowledge that their personal and professional backgrounds shaped the analytical perspective adopted in this study. Approximately half of the participants were recruited from existing research databases and some had prior contact with members of the research team through previous studies and community engagement activities. These relationships were considered throughout recruitment, interviewing, and analysis. During interviews, care was taken to avoid assumptions of shared understanding, and participants were encouraged to explain experiences in their own words. During analysis, reflexive discussions considered how familiarity with some participants may have shaped interpretation of the data. Longstanding involvement with a local Breathe Easy support group, together with sustained engagement with individuals living with COPD before and during data collection, contributed to trusting researcher–participant relationships, which may have enhanced the depth and openness of disclosures. SG further recognises that both lived experience of depression and professional experience within NHS clinical settings may have informed the dynamics of participant interactions and influenced the interpretation of psychologically oriented narratives. SG’s positionality as a clinician-researcher offered advantages, such as sensitivity to patient experiences and facilitated rapport, while also carrying the risk of interpreting accounts through a clinical lens. This dual role necessitated continuous reflexive awareness to balance empathy with analytic distance and ensure that participants’ voices, rather than professional assumptions, informed the analysis. To mitigate potential bias, SG employed open, non-leading questioning, maintained clear boundaries between personal experience and analytic decision-making, and engaged in regular supervisory discussions to critically examine emerging interpretations. Reflexive attention was also given to differences in rapport between participants previously known to the researcher and those with no prior contact, recognising that these dynamics may have shaped the richness and texture of the data. At the time of interviewing, SG held an MSc in Psychology. Although younger than the participants, SG’s White British background aligned with that of most individuals in the sample. SG had received formal qualitative methods training during both BSc and MSc studies and had previously used reflexive thematic analysis in dissertation research, providing a strong methodological foundation for the analytic approach undertaken in this study.

## Results

Fifteen semi-structured interviews were conducted to explore patients' experiences of COPD, with particular focus on symptom burden, treatment experiences, and self-management practices. The interviews also explored participants' perceptions of current care and the strategies they employed to manage the ongoing challenges associated with living with COPD. Reflexive thematic analysis generated three themes that captured patterns of shared meaning across participants' accounts: (1) Interconnected symptom experiences, (2) Negotiating treatment uncertainty and burden, and (3) Becoming the expert in managing COPD. Together, these themes illustrate how participants experienced COPD not as a collection of isolated symptoms, but as a condition requiring continual adaptation, evaluation, and self-management.

### Theme 1: Interconnected symptom experiences

Participants described COPD symptom burden as extending beyond breathlessness alone. Accounts frequently reflected an interconnected network of breathlessness, sleep disruption, fatigue, reduced activity, and social restriction. Rather than occurring independently, these symptoms were experienced as collectively shaping daily decision-making and quality of life.

#### Sub-theme 1: Breathlessness as an unpredictable threat that shapes daily choices

Most patients reported experiencing breathlessness that significantly affected their daily lives, particularly by limiting their mobility and reducing their energy levels. Reduced mobility meant that many were unable to engage in leisure activities such as playing sports or walking longer distances, resulting in a noticeable decline in overall day-to-day functioning. Patients in the later stages of COPD described becoming breathless with even minimal movement. Some struggled so intensely to draw breath that they became completely sedentary until their breathing stabilised. Beyond restricting physical activity, many reported that breathlessness also drained their energy and left them fatigued. For some, regular ambulatory oxygen was necessary to maintain mobility and support breathing during even basic tasks, including conversation.

*“The main one being breathlessness…When I move at all, you can see I’ve got oxygen on which I use when I move about, but also, for example, if I’m taking part in a conversation, I need that” (Melissa)*

*“Breathing lets you down every time. I start doing a bit of stuff, and then the next thing is you can't breathe, but then it puts you into a frame where you don't think you don’t want to do that, then why should I put myself in that frame” (Marcus)*

These accounts suggest that breathlessness was experienced not simply as a symptom, but as an unpredictable threat that restricted independence and altered how participants engaged with everyday life.

Participants also described emotional consequences associated with breathlessness, particularly fear, anxiety, and uncertainty surrounding episodes of severe respiratory discomfort. For some, concerns about struggling to breathe became especially prominent during the night.

*“I am fearful of going to bed at night-time because I know I am going to be gasping but I do use ambulatory oxygen which I know I should only use the oxygen to help me last longer or whatever task I might be doing but if I plug it in and have a really big gasp of oxygen therapy when I have been doing quite a lot so whether I am doing the right thing but it seems to help” (Tracy)*

Breathlessness therefore appeared to influence both physical functioning and psychological wellbeing, contributing to broader experiences of vulnerability and loss of control.

#### Sub-theme 2: Poor sleep amplifies the burden of living with COPD

Participants frequently described poor sleep as a significant but often overlooked consequence of COPD. Although breathlessness commonly disrupted sleep, participants also described coughing, mucus production, anxiety, and general discomfort as factors contributing to fragmented sleep.

*“I cough at night in bed and I said I don't think I do and he said well do you wake up at night and I said yes and he said well that might be because you are coughing but I'm not coughing the rest of the night I don't think it is actually I don't think it is but yeah my sleep is poor I'm either awake I sleep till four and then I'm awake or I wake up one after going to bed at 10:30 and then I'm awake until four or five yeah” (Yvonne)*

Poor sleep was not described as an isolated problem. Rather, participants portrayed sleep disruption as amplifying the burden of COPD by worsening fatigue, reducing motivation, lowering activity levels, and increasing social withdrawal.

*“I do fall asleep I I've been known to do it through probably started in my 30 s if I was not actually taking part in something I was just being a watching or something like being in a church or something and suddenly falling asleep but I do that a lot more now especially in the evening so I guess that's me catching up on sleep or something but I will suddenly and I won't know I’m falling asleep it is only when I wake up that I realise that I've been asleep” (Pat)*

*“I get very tired yeah I just have no energy so I’ll go out and do about 40 minutes or so then I have to come back in I can do longer than that but then I’m really awful later on so and again it is thinking about other things that I want to do later shall I go for a walk cook tea you know and all of those things need energy to do them so and some days I am fighting and doing them regardless but other days I’m not” (Suzanne)*

Participants often described a compounding relationship between poor sleep, fatigue, and reduced activity, whereby sleep disruption diminished their capacity to engage in daily tasks, which in turn contributed to feelings of frustration, dependence, and restricted social participation. Taken together, participants' accounts suggested that symptom burden was experienced as interconnected and mutually reinforcing rather than as discrete clinical symptoms occurring independently.

### Theme 2: Negotiating treatment uncertainty and burden

Participants' discussions of medication reflected an ongoing process of balancing symptom relief against treatment burden. Rather than viewing medications as straightforward solutions, participants frequently described uncertainty regarding effectiveness, concerns regarding side effects, and frustration associated with repeated treatment changes.

#### Sub-theme 1: Accepting side effects as the price of symptom control

Participants described a range of medication-related side effects, including tremors, palpitations, sleep disturbances, oral thrush, and concerns regarding longer-term health consequences. Despite these challenges, many participants continued using medications because symptom control remained their primary concern.

*“I'm going to do a cost benefit analysis haven’t I well you know judging by the disease progression in the last four years and I can see what that is then what I want potentially the next four years if I've got four years but I want to be like yeah because it's not it's not quantity of life for me its quality of life really that matters and it's difficult weighing it all up I I put off going on statins for a long time…… if I want to reduce my risk of stroke or heart disease then it makes sense to take them and it has done the trick and it has lowered my cholesterol so that's a huge help really so it is weighing that up all the time I think” (Aimee)*

Accounts frequently reflected a process of weighing immediate symptom relief against undesirable side effects. Rather than viewing treatment decisions as clear-cut, participants described continually reassessing what they were willing to tolerate in exchange for maintaining daily functioning and quality of life. One participant described using a bronchodilator up to twelve times per day during periods of significant symptom burden.

*“I would have I don't know probably about 12 doses a day on average and that would be on good days let alone bad days these days I'm probably only having one or two doses every few days of the Terbutaline I'm starting now to use it a bit more frequently because I need it more so I then also more or less around” (Jane)*

Such accounts highlighted the extent to which participants adapted medication use in response to ongoing symptoms and changing circumstances.

#### Sub-theme 2: When treatment fails to match lived experience

Participants frequently questioned whether medications were providing meaningful benefit. Although participants generally adhered to prescribed treatments, several reported difficulty recognising improvements in symptoms following medication use.

*“I don't feel any difference and I take the medication (laughter) I go and do something and I still think what is the point in that and I still take it because you know it might be worse if I don't take it and I am on heart medication as well blood thinning tablets I have enough tablets do I like taking them no but I do I do as I am told it's meant to assist me but I don't… yeah absolutely and as I say if I stop taking the inhalers I don't think I would feel any different that is how they affect me I can't turn around after taking them and think oh crikey I can go for 10 minutes and run up the stairs and pick up some washing and bring it down again I still have to go up the stairs and get my breath back and then walk down the stairs” (Patrick)*

Participants often contrasted medical expectations of treatment effectiveness with their own lived experiences of continuing breathlessness and functional limitation. This mismatch appeared to contribute to frustration, uncertainty, and diminished confidence in treatment. Medication changes were often experienced as disruptive events requiring considerable adaptation.

*“I have just been having one issue and they are all so interrelated I seem to have been losing excessive protein from my kidneys so they changed my medication which included a diuretic to stop my ankles from swelling because my circulation is not very good there and we changed over to another medicine which should have been more helpful in there controlling the output of the kidneys but now we have now been able to stabilise that so we are changing back to the diuretic and when I was off the diuretic the ankles on my legs swelled really badly” (Tracy)*

Taken together, these accounts suggest that medication management was experienced not as a routine aspect of care but as an ongoing process of uncertainty, adjustment, and negotiation.

### Theme 3: Becoming the expert in managing COPD

Participants consistently portrayed themselves as active rather than passive recipients of care. Living with COPD required continual problem-solving, adaptation, and learning, leading many participants to develop considerable expertise regarding their own symptoms, capabilities, and management strategies.

#### Sub-theme 1: Developing personalised strategies for daily living

Participants described employing a range of practical strategies designed to preserve independence and manage everyday challenges. These included pacing activities, planning journeys, using mobility aids, and adopting assistive devices to minimise physical strain.

*“yes I do and I have every device known to man to help me I have a stairlift I have a car with a hoist in the back so I can put a portable scooter and I have two scooters and I live on a bit of a slight hill and I am not far from ________ in _______ I could happily walk to _______ but I couldn't walk back because of the hill up to my house so those are the main restrictions but yes I do have to plan my route very carefully if I have to visit anywhere if I am booking a hotel I have to make sure you know there are accessible rooms and stuff like that” (Teresa)*

Rather than representing simple solutions, these strategies reflected ongoing efforts to retain control over daily life despite progressive limitations. Participants frequently described adapting routines, environments, and expectations to maintain independence. Such adaptations reflected resilience and agency but also highlighted the substantial work required to manage COPD outside clinical settings.

#### Sub-theme 2: Seeking partnership in care and research

Participants expressed a strong desire to contribute actively to treatment decisions and healthcare planning.

*“I like to do my own research I like to be involved in my care as you can imagine I like to be involved in my care I like to have somebody who I can work with to do that the Dr we have a superb in house pharmacist here he is absolutely brilliant so yeah so medication is not only for the COPD but for the comorbidities as well” (Yvonne)*

Many participants viewed lived experience as a valuable source of expertise and expressed frustration when opportunities for involvement were limited. Several participants also expressed willingness to engage in future research.

*“Anything is better than this, but I have never been asked to participate in anything” (Yvonne)*

These accounts suggest that participants viewed involvement in care and research not simply as opportunities to receive support, but as ways of exercising agency, contributing knowledge, and helping improve outcomes for others living with COPD. Across participants' accounts, self-management emerged as an essential and often under-recognised component of COPD care. Participants described becoming experts in navigating the realities of COPD, drawing on both formal healthcare and personal experience to manage the complex challenges of everyday life.

## Discussion

This study provides a detailed, patient‑centred account of COPD symptom burden and management, highlighting the complex, interconnected nature of lived symptom experience and the limitations of current treatment approaches. Rather than describing symptoms in isolation, participants' accounts illustrated how breathlessness, sleep disruption, fatigue, treatment experiences, and self-management behaviours were experienced as interconnected aspects of daily life. The findings suggest that participants experienced breathlessness as a central, organising symptom that was closely associated with sleep disruption, fatigue, reduced physical activity, and social withdrawal. While previous research has consistently identified dyspnoea as a hallmark symptom of COPD (O’Donnell & Neder, 2020), the present findings extend this literature by illustrating how breathlessness is not experienced in isolation but is embedded within a broader network of interconnected symptoms that progressively constrains daily functioning and quality of life.

The interaction between breathlessness and sleep disturbance is particularly noteworthy. Participants frequently described poor sleep, daytime fatigue, and breathlessness as closely interconnected experiences. Although the present study cannot establish causal relationships between these symptoms, participants often portrayed them as mutually influential, with poor sleep reducing energy for daily activities and breathlessness contributing to sleep disruption and anxiety. Similar associations between sleep problems, fatigue, and diminished quality of life have been reported in COPD populations (Shorofsky et al., 2019; Zeidler et al., 2018). Participants in the present study similarly described sleep disruption as closely linked to fatigue, reduced activity, and difficulties engaging in everyday tasks. Importantly, participants' accounts suggest that symptom burden may be experienced as cumulative rather than additive, whereby challenges associated with one symptom can intensify the impact of others. This interpretation aligns with Symptom Management Theory (Dodd et al., 2001), which conceptualises symptom experience as a dynamic and interacting process rather than a series of isolated clinical events.

From an applied perspective, these findings support greater attention to the broader symptom experience of COPD within routine care. Despite engagement with guideline-directed pharmacotherapy, participants frequently reported ongoing breathlessness, fatigue, and sleep disruption, echoing wider evidence that symptom burden often persists despite treatment (Celli, 1995; Hillas et al., 2026). While this study did not examine healthcare decision-making directly, participants' accounts suggest that some aspects of symptom burden, particularly sleep quality, fatigue, and emotional responses to breathlessness, may receive less attention than they themselves considered important. These findings highlight the value of incorporating routine discussions of broader symptom experiences alongside traditional clinical assessments.

The findings also identify sleep disturbance as an important component of participants' lived experiences. While PR remains one of the most effective non-pharmacological interventions for COPD (McCarthy et al., 2015; Osadnik & Singh, 2019), participants' accounts suggest that sleep-related difficulties may warrant greater consideration within COPD management pathways. The present findings do not evaluate the effectiveness of sleep-focused interventions. However, future research may benefit from exploring whether interventions targeting sleep quality could contribute to improving broader aspects of symptom burden, including fatigue and daily functioning.

Psychological factors were also highly salient, particularly anxiety associated with breathlessness and fears relating to the prospect of struggling to breathe. These findings are consistent with previous qualitative work highlighting emotional distress associated with COPD symptom experiences (Barnett, 2005; Shackell et al., 2007) and quantitative evidence demonstrating elevated rates of anxiety among individuals living with COPD (Anlló et al., 2022; Siraj, 2025). Participants' accounts suggest that emotional responses were closely intertwined with physical symptoms, particularly during episodes of significant breathlessness and at night. Within Leventhal's Common-Sense Model, such responses may be understood as emotional illness representations that influence coping strategies and behavioural responses.

However, the present study adds depth by linking these emotional responses directly to symptom experience and daily functioning. Leventhal’s Common‑Sense Model (2016) suggests that fear responses can be understood as part of patients’ emotional illness representations, which in turn shape coping behaviours, including avoidance of activity and disrupted sleep patterns. These findings highlight the importance of recognising the emotional dimensions of breathlessness within COPD care and provide support for future research exploring how psychological support may be integrated within existing management pathways. These findings support further exploration of how psychological support may be incorporated into COPD care pathways and pulmonary rehabilitation programmes.

Medication-related challenges further illustrate the complexity of COPD management. Participants described an ongoing process of balancing symptom control against the burden of treatment side effects, consistent with previous work exploring treatment burden and adherence in COPD (Restrepo, 2008; van Boven et al., 2016). Notably, participants often evaluated treatment effectiveness through their ability to perform everyday activities rather than through clinical indicators of disease management. This appeared to contribute to frustration when medications were perceived as offering limited improvement in symptoms or quality of life. Several participants also described independently modifying medication use in response to side effects or perceived need. While inhaler technique and objective treatment adherence were not explored within the present study, these findings highlight the importance of ongoing patient-clinician dialogue regarding treatment expectations, side effects, and symptom experiences. Rather than reflecting simple non-compliance, participants' accounts suggest that medication use often involved active interpretation, judgement, and adaptation based upon lived experience.

The prominence of self-management strategies further highlights the active role participants played in navigating their condition. Participants described pacing, assistive devices, route planning, self-directed learning, and extensive personal adaptation in response to changing symptom burden. These findings align with previous work emphasising patient agency in chronic disease management (Wodskou et al., 2014). However, the present findings extend existing literature by illustrating how self-management was experienced not simply as a clinical expectation, but as an essential component of maintaining independence, social participation, and quality of life. Participants frequently described becoming highly knowledgeable about their condition through lived experience, often positioning themselves as experts in managing the realities of everyday COPD. These findings reinforce the importance of collaborative and patient-centred approaches to care, in which clinical expertise is combined with patient experience and preferences when developing management strategies.

An additional finding was participants' strong desire for greater involvement in both healthcare decision-making and research. Several participants viewed lived experience as valuable expertise and expressed frustration when opportunities for participation were limited. Importantly, these findings should be interpreted within the context of the study sample. Many participants were already connected to support organisations, previous research databases, or patient communities, which may have increased their awareness of and interest in research participation compared with the wider COPD population. Nevertheless, their accounts highlight the potential value patients place on meaningful involvement in shaping future research and service development.

The primary contribution of this study lies not in identifying individual symptoms or management challenges, which have been described previously, but in demonstrating how participants experienced symptom burden, treatment challenges, and self-management behaviours as interconnected and mutually influential aspects of everyday life. By examining these experiences together rather than separately, the findings provide a more integrated understanding of living with COPD and highlight the substantial adaptive work undertaken by patients outside formal healthcare settings.

### Limitations

Several limitations should be considered. The sample was relatively small and demographically homogeneous (predominantly older White British individuals), limiting transferability to more diverse populations. Furthermore, many participants were recruited through support organisations or previous research databases, and approximately half had previously participated in research. Consequently, participants may have been more engaged in self-management, healthcare discussions, and research activities than the wider COPD population. Recruitment occurred through multiple routes, including participant databases and community advertisements. As the number of individuals approached and declining participation was not systematically recorded, recruitment yield and refusal rates cannot be reported. This limits assessment of potential participation bias. Disease severity was not formally assessed, restricting interpretation of how experiences may differ across disease stages. The use of online interviews may have excluded individuals with limited digital access or digital literacy, potentially biasing the sample towards more engaged participants. Additionally, prior relationships between the researcher and some participants may have influenced the depth and nature of disclosures, although reflexive practices were employed throughout the study to mitigate this influence.

### Future research

Future research should explore symptom burden across more diverse populations, including individuals from ethnically diverse backgrounds, younger age groups, and those less engaged with support services or research activities. Incorporating objective indicators of disease severity may allow exploration of how symptom experiences vary across COPD stages. Future studies should also investigate the relationships between sleep disturbance, fatigue, breathlessness, and psychological wellbeing, as these emerged as important interacting experiences within participant accounts. Finally, intervention research that is informed by patient priorities and experiences may help develop approaches that better reflect the realities of living with COPD.

## Conclusion

This study suggests that people living with COPD experience symptom burden, treatment challenges, and self-management as interconnected aspects of everyday life. Participants described breathlessness, sleep disruption, and fatigue as closely related experiences that influenced daily functioning, while medication-related challenges and substantial self-management demands further shaped living with the condition. Together, these findings provide a more integrated understanding of COPD lived experience and highlight the importance of recognising patient perspectives when designing and delivering person-centred care.

## Data Availability

The datasets generated and analysed during the current study are not publicly available due to the sensitive and potentially identifiable nature of the qualitative data but are available from the corresponding author on reasonable request, subject to appropriate ethical and data protection approvals.
